# *Porphyromonas gingivalis* deteriorates autism spectrum disorders by disturbing the gut and oral microbiota

**DOI:** 10.3389/fmicb.2025.1579128

**Published:** 2025-08-06

**Authors:** Bosheng Li, Yanan Qiao, Yongming Li

**Affiliations:** ^1^Shanghai Engineering Research Center of Tooth Restoration and Regeneration, Tongji University, Shanghai, China; ^2^Tongji Research Institute of Stomatology, Tongji University, Shanghai, China; ^3^Department of Orthodontics, Stomatological Hospital and Dental School, Tongji University, Shanghai, China

**Keywords:** *Porphyromonas gingivalis*, autism spectrum disorder, 16S rRNA, intestinal flora, oral flora

## Abstract

Autism spectrum disorder (ASD) significantly impairs socialization and communication, posing a major societal challenge due to limited understanding of its pathogenesis and lack of effective treatments. Recent studies have shown an imbalance in the oral and intestinal microbiota of individuals with ASD, which may exacerbate ASD symptoms. In this study, we successfully established an ASD mouse model induced by *Porphyromonas gingivalis* (*Pg*) solution. Bio-behavioral experiments, including the elevated plus maze test, demonstrated that Pg. induced anxiety-like behaviors in mice. Analysis of oral and intestinal microbiota revealed significant alterations in microbial richness, diversity, and evenness in *Pg*-treated mice, indicating that Pg. disrupted the normal bacterial community structure and function. Subsequent 16S rRNA sequencing showed increased abundance of amino acid metabolism pathways in *Pg*-intervened mice, highlighting the close link between bacterial community function and carbohydrate, amino acid, and nucleotide metabolic pathways. These findings provide promising clinical targets for ASD treatment and offer insights into developing new therapeutic strategies.

## 1 Introduction

Autism spectrum disorder (ASD) is a group of developmental and phenotypic diseases that relate to cause serious problems with behavior, socialization, and communication (Maenner et al., 2023). ASD has become a more and more serious public concern with its increasing incidence all over the world. Currently, about one in 100 children is diagnosed with ASD, and this disorder is 3.8 times as prevalent among boys (4.3%) as among girls (1.1%) (Maenner et al., 2023). So, the study of ASD is of both great importance and research significance. However, current understanding about its complicated pathogenesis is far from adequate, which is highly relevant to its potential therapeutical targets finding and treatment strategy developing.

There is a tight link between flora imbalances and mental disorders. On the one hand, a growing body of research indicates that the gastrointestinal (GI) microbiota, a vital gut-brain axis mediator, is crucial to human psychological disorders such as autism (Liu et al., 2022). On the other hand, in people with ASD, gastrointestinal issues are prevalent (Wang et al., 2011). For example, A growing body of research using germ-free mice indicates that total gut bacterial elimination reduces blood-brain barrier integrity, lessens behaviors associated with anxiety and depression (Diaz Heijtz et al., 2011), impairs social cognition (Desbonnet et al., 2014), and causes molecular alterations in the prefrontal cortex, hippocampus, and amygdala (Liu et al., 2020). More and more studies favored the opinion that microbiota distribution differences make a big difference in the development of ASD, such as *Bifidobacterium*, *Prevotella*, *Sutterella*, *Ruminococcus*, etc. Autism’s incidence and progression are mainly influenced by abnormal microbiome.

Recently, Stephen S Dominy et al. reported that Pg. was found in the brain of Alzheimer’s disease (AD) patients and presented sound evidence for its causation for this disease, showing an attractive development prospect, accompanied by a series of studies to confirm this phenomenon or elaborating the mechanism (Li et al., 2017). As a well-characterized and distinct bacterium, Pg. can enhance dysbiosis, induce chronic inflammation, contribute to the pathogenesis of periodontitis, cause tissue damage, and invade the weakened epithelial cell layers, leading to systemic diseases (Howard et al., 2021). Studies involving *Pg*, however, revealed an apparent paradox: despite being closely linked to inflammatory diseases, the bacteria did not appear to be a powerful inflammatory inducer (Darveau et al., 2012). Meanwhile, ASD and AD are neurodevelopmental and neurodegenerative disorders affecting two opposite ends of life span, and they share many signs and symptoms such as poor cognitive skills, anxiety and depression, neurological problems, restlessness and disturbed sleep, speech impairment and language problems (Nadeem et al., 2021). Moreover, a variety of critical genes including MECP2 (Methyl-CpG Binding Protein 2) (Wen et al., 2017), PTEN (phosphatase and tensin homolog) (Zhou and Parada, 2012), APOE (apolipoprotein E) (Husain et al., 2021), AKAP9 (A-kinase anchoring protein 9) (Poelmans et al., 2013), SLC6A4 [solute carrier family 6 (neurotransmitter transporter, serotonin), member 4] (Calabrò et al., 2020) and etc. have been reported to play vital roles in both ASD and AD. As a result, there is an inherently strong connection between AD and ASD, suggesting that therapy approaches that are potentially effective for AD may also be effective for ASD. Drawing from the aforementioned facts and the absence of study reports about the connection between *Pg.* and ASD, we hypotheisze that *Pg.* plays a crucial role in the development of ASD and could serve as a viable therapeutic target.

In this study, the implications of Pg. on the ASD phenotypes were evaluated in mice by detecting the behavioral performance of *Pg*-treated mice compared to Con mice, with the primary mechanism explored. Next, the underlying mechanism was further investigated via gut-oral microbial diversity analysis, presenting several useful clues and potential targets for interpreting the unveiled correlation between Pg. and ASD. This work provides new evidence for the vital role of *Pg.* in the development of autism, and provide a good reference for the diagnosis and treatment of this serious disease.

## 2 Materials and methods

### 2.1 Animal housing and grouping

Mice were housed in a specific pathogen-free (SPF) facility with *ad libitum* access to food and water. They were divided into two time points (T0 and T1, samples collected before the 3-month Pg/Vehicle treatment were designated as T0, while those collected after the treatment were called T1), with 10 mice in the CON group and 15 in the Pg. group. The Pg/Vehicle treatment was carried out over a span of 3-months to ensure thorough remodeling of the oral-gut microbiota (Liu et al., 2024). Pg. group mice received oral gavage of Pg. (10^∧^9 CFU). The Pg. group was administered 0.1 ml of bacterial suspension (ATCC33277) containing 10^∧^9 CFU + 2% carboxymethyl cellulose, while the CON group was given 0.9% saline solution containing 2% carboxymethyl cellulose. Gavage was performed three times weekly for 12 weeks. All procedures adhered to scientific and humane principles, using a specialized gavage needle to minimize discomfort and harm to the mice.

### 2.2 Elevated plus maze

To minimize stress, the laboratory environment was kept quiet on the day of the experiment, and the maze was cleaned to remove residual odors. Mice were placed in the center of the maze, facing the open arm, and allowed to explore freely. Open arm activity was defined as the time when the animal’s limbs were over 80% of the body in the open arm. The video tracking system recorded the number and duration of entries into the open arms, as well as the total movement time. Data were analyzed using behavioral software. After the experiment, mice were returned to their breeding cages.

### 2.3 Three-chambered social approach

The three-chambered social approach test assessed mouse sociability using a rectangular apparatus (60 cm × 40 cm × 22 cm). The experiment included habituation and social interaction phases. During habituation, mice freely explored all chambers for 10 min. In the first interaction phase, an unfamiliar mouse (Stranger 1) and an inanimate object were placed in side chambers, and the test mouse explored for another 10 min. One hour later, a second unfamiliar mouse (Stranger 2) replaced the object, and a 5-min observation recorded interaction times with Stranger 1 and Stranger 2. Chamber stay time, interaction duration, and entry frequency were documented.

### 2.4 Self-grooming test

The self-grooming test was executed following established protocols. Individual mice were introduced to the standard grooming test cage. Once the mice had acclimated to the environment for 10 min, an observation procedure was initiated to monitor and document their grooming behavior for an additional 10 min. A trained, blinded observer assessed the videos, unaware of the mice’s drug treatment, to score the grooming activities.

### 2.5 Marble burying

Marble burying (MB) was performed in a normal cage bottom (Shanghai Youer Equipment Scientific Co., Ltd.) with floor area of 75 in 2 filled with 3–4 cm of fresh, autoclaved wood chip bedding. Mice were first habituated to the cage for 10 min. Then place 20 glass beads, each with a diameter of 10 millimeters, evenly on the bedding material, arranged in a 5 × 4 pattern. Return the experimental mice to the cage and observe for 30 min, recording the number of buried glass beads (beads are at least two-thirds covered by the bedding material). After each experiment, smooth the bedding material again for reuse.

### 2.6 Open field testing

Open field testing (OFT) was performed in 50 × 50 cm^2^ white box, recorded using an overhead camera, and tracked and analyzed using the software package. Prior to testing, the arena was disinfected using 70% ethanol and finally water. Mice were then introduced to the arena and allowed to explore for 10 min while tracked. The total distance traveled, and the number of entries and time spent in a 30 × 30 cm^2^ center square, were analyzed by the software.

### 2.7 Light-dark box test

Adjust lighting intensity to 200–400 lux in bright areas and below 5 lux in dark areas. Ensure the camera system functions properly to accurately capture mouse behavior. Allow mice 30 min to adapt to the environment before starting the experiment to minimize stress. Place mice gently in the center of the bright area, facing away from the dark room opening. Record the latency to enter the dark room and observe for 5–10 min, noting the number of crossings and duration in the bright area.

### 2.8 Sequence processing analysis

This study employed Illumina paired-end sequencing to analyze community DNA fragments. After quality control with DADA2, sequences were identified as amplicon sequence variants (ASVs) or operational taxonomic units (OTUs). Statistical analysis confirmed that sequence lengths aligned with the expected range, with no anomalies. We analyzed 50 samples at two time points (T0 and T1), with 10 in the CON group and 15 in the experimental group. The sequencing region was 338F_806R, with an insert fragment length of 468 bp. Paired-end 250 (PE250) sequencing generated 6,519,144 raw sequences and a total base count of 1,629,786,000. After quality control and assembly, 3,259,572 optimized sequences were obtained, averaging 426 bp in length. For the oral flora, we tested 20 samples (10 per group). After quality filtering, denoising, and chimera removal, the CON group averaged 50,689.3 sequences per sample, while the experimental group averaged 59,022.7 sequences per sample.

### 2.9 Species composition analysis

By statistically processing the rarefied ASV/OTU table, based on the results of sequence species taxonomic annotations and the selected samples, we count the number of taxonomic units at seven classification levels: kingdom, phylum, class, order, family, genus, and species. By statistically analyzing the feature table after removing singletons, utilizing stacked bar charts, heatmaps, and Venn diagrams to visualize the compositional distribution of samples at the phylum, class, order, family, genus, and species taxonomic levels.

### 2.10 Species difference and indicator species analysis

Alpha diversity was analyzed using indices such as Ace, Chao, Shannon, Simpson, Sobs, and Coverage, calculated based on observed OTUs.

Rarefaction Curve: Constructed using alpha diversity indices of samples at various sequencing depths, the rarefaction curve plots the number of sequences sampled against species (ASV/OTU) counts or diversity indices.

Abundance Rank Curve: This curve ranks ASVs/OTUs by abundance along the horizontal axis, with their abundance values on the vertical axis. The curve reflects the distribution of high-abundance and rare ASVs/OTUs, plotted using R after log transformation of abundance values.

Gut Microbiome Health Index (GMHI): GMHI analysis involves gathering metagenomic data from fecal samples, including healthy and diseased individuals. After quality control, taxonomic analysis identifies differentially abundant microbial species. The GMHI is calculated based on the relative abundance of these health-associated species.

Intestinal Dysbiosis Index (MDI): MDI assesses gut microbiota imbalance using abundance ratios, differences, or linear regression of specific taxa.

### 2.11 Species and functional difference analysis

Utilizing statistical test methods such as the Kruskal-Wallis test, Wilcoxon rank sum test, PERMANOVA and Student’s *t*-test, species, functions, or gene abundance data annotated from databases like Metacyc, KEGG (Kyoto Encyclopedia of Genes and Genomes) and COG (Clusters of Orthologous Groups) are subjected to hypothesis testing to assess the differences in microbial community species, functions, or gene abundance between different groups. This process evaluates the significance level of species, functional, or gene abundance differences, and identifies species, functions, or genes with significant differences between groups.

### 2.12 LS-MS analysis

The concentration of short-chain fatty acids (SCFAs) in serum was determined by LC-MS. Serum samples (150 μL) were mixed with acetonitrile solution containing internal standards, subjected to ultrasonic extraction and centrifugation, and the supernatant was collected for derivatization before analysis by LC-MS.

## 3 Results

### 3.1 Pg. oral administration exacerbated ASD-like behavior

**FIGURE 1:**
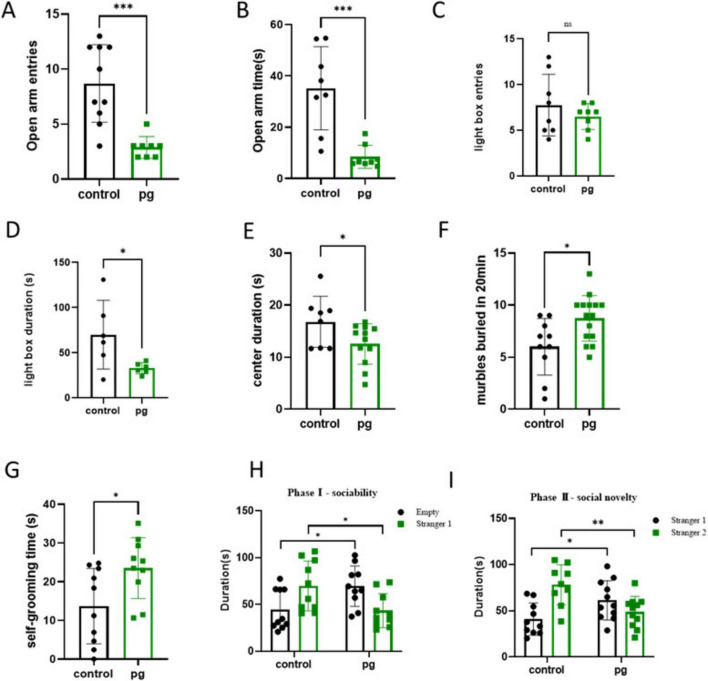
Behavioral tests of the Con and *Pg*-treated mice. **(A)** Quantity of entries into the open arm. **(B)** Time to enter the open arm. **(C,D)** The light–dark box test. **(E)** Open field test. **(F)** Marble Burying Test. **(G)** Self-grooming test. **(H,I)** Three-chambered social approach. *p < 0.05, **p < 0.01, ***p < 0.001.

The study tested CON and Pg. group mice for ASD-related behaviors. Pg. oral administration worsened anxiety, repetitive stereotyping, and social behaviors in mice. Anxiety, a key ASD trait, was assessed using elevated plus maze, light-dark box, and open field tests. Pg. treated mice entered the open arm less frequently (Figure 1A) and spent significantly less time in the open arm (Figure 1B), indicating a reduction in mouse activity. The light-dark box test showed a tendency of decreased activity frequency and indicated significantly less time in bright areas, suggesting reduced exploration behaviors (Figures 1C,D). The open field test showed Pg. mice spent less time in the central area, correlating with increased anxiety (Figure 1E). High anxiety often leads to repetitive behaviors, measured by marble burying and self-grooming tests. Pg. treated mice buried more marbles, indicating heightened repetitive behaviors (Figure 1F). Increased self-grooming, a self-soothing mechanism, was also observed in Pg. mice, reflecting elevated anxiety (Figure 1G). Social activity was assessed using a three-chambered test. Normal mice preferred interacting with unfamiliar mice (Stranger 1) over an empty cage and showed novelty preference by interacting more with a new mouse (Stranger 2). Pg. mice spent more time with the empty cage and less with Stranger 1 initially. In the second stage, they spent more time with Stranger 1 but less with Stranger 2, indicating impaired social novelty and memory (Figures 1H,I). Furthermore, social preference indices was used to enhance the experimental results (Supplementary Figures S1C,D). These findings suggest Pg. oral administration reduces social willingness and novelty-seeking behavior.

### 3.2 Pg. administration triggered changes in gut flora in mice

#### 3.2.1 Pg. oral administration reduces the diversity of intestinal flora of mice

**FIGURE 2:**
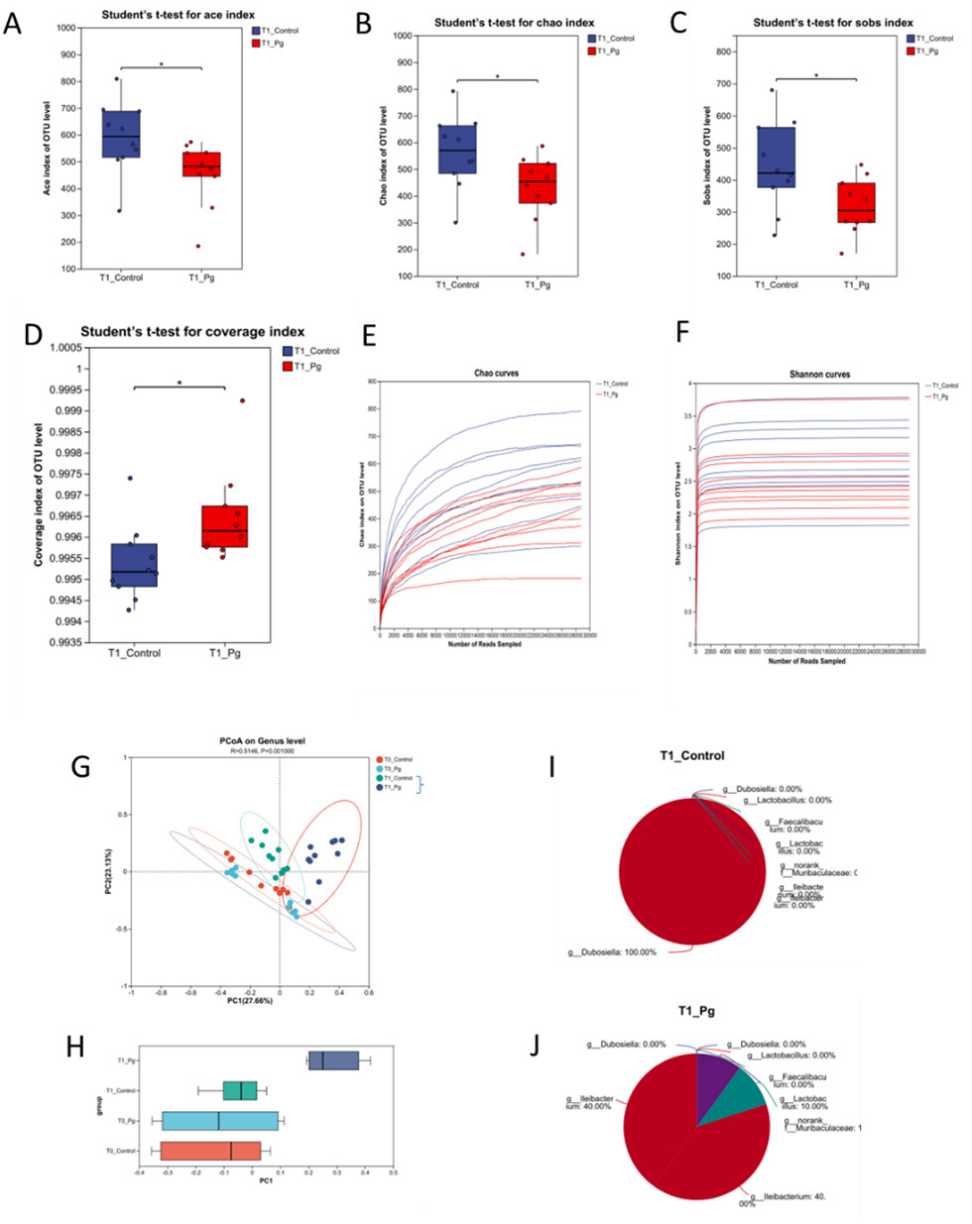
Alpha and beta diversity and Microflora typing analysis. **(A–D)** Alpha diversity analysis for comparison between Con and Pg. groups. **(E,F)** The dilution curves based on Chao and Shannon indexes. **(G,H)** First two axes of a principal coordinate analysis (PCoA) of Bray Curtis distances from Con and Pg. mice at the genus level. P indicates the significant differences between T1 CON and T1 Pg. groups. Points of different colors or shapes represent samples of different groups. **(I,J)** Microflora typing analysis. *n* = 10 for each group. *p < 0.05, **p < 0.01, ***p < 0.001.

Following Pg. oral administration, 50 fecal samples were collected for 16S rRNA sequencing. Optimized sequences averaged 426 bp, with lengths ranging from 200 to 452 bp, indicating well-distributed data (Supplementary Figures S2A,B). A heatmap and cluster tree revealed distinct clustering, with T0 groups partially overlapping and T1 groups fully separated, suggesting Pg. significantly altered gut flora composition (Supplementary Figures S2C,D). Environmental factors also influenced flora structure over time, prompting focus on T1 CON and T1 Pg. groups for further analysis. Alpha diversity indices (Ace, Chao, Shannon, Simpson, Sobs, coverage) were calculated at the OTU level (Figures 2A–F and Supplementary Figures S3A–F). Pg. oral administration Ace, Chao, and Sobs indexes, indicating decreased microbial richness, diversity, and uniformity (Figures 2A–D). Dilution curves based on Chao and Shannon indices confirmed adequate sequencing depth, with Pg. group values lower than CON, reflecting reduced species richness (Figures 2E,F). Beta diversity analysis used PCoA of Bray-Curtis distances and PERMANOVA, revealing significant differences in bacterial community structure between T1 CON and T1 Pg. groups (Figures 2G, H and Supplementary Figures S3G, H). Top 8 genus analysis (CH index peak at K = 8) showed Ileibacterium and Ileibacterium_1 predominant in T1 Pg, while Dubosiella was most abundant in T1 CON (Figures 2I,J).

#### 3.2.2 Pg. oral administration altered the gut microbiome and impaired gut microbiota health

**FIGURE 3:**
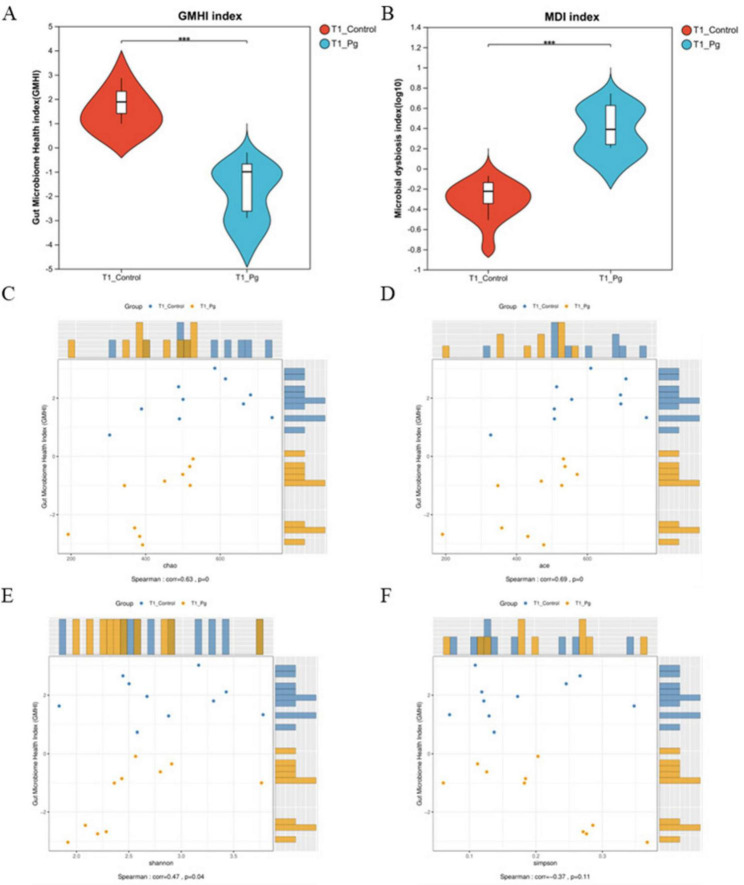
Gut flora health status estimation. **(A)** Gut microbiome health index analysis (GMHI). **(B)** Intestinal dysbiosis index (MDI) analysis in genus level. Using the Wilcoxon two-tailed rank-sum test, the MDI score represents the degree of microflora disorder severity. **(C–F)** Correlation analysis between GMHI and Alpha diversity index. The vertical coordinate is the GMHI index and the horizontal coordinate is the alpha diversity index. *p < 0.05, **p < 0.01, ***p < 0.001.

After detecting the OTU levels of different groups, we continue to calculate the Gut microbiome health index analysis (GMHI) to further evaluate the health status of the mice. The data showed that the GMHI of Pg. group was markedly lower than that of the CON group, indicating a significant health deterioration in the Pg. treated mice (Figure 3A). Meanwhile, there was a significant difference between the CON and Pg. groups in terms of the gut dysbiosis index (MDI), suggesting that the Pg. group encountered serious imbalance of gut microflora (Wilcoxon two-tailed rank-sum test, Figure 3B). Additionally, the GMHI’s stratification power for health between treatment groups was significantly stronger than each alpha diversity index, as demonstrated by the correlation analysis that follows, which also shows that the GMHI was significantly consistent with the distribution of the Chao, Ace, and Shannon indices (Figures 3C–F).

#### 3.2.3 Analysis of intestinal species composition and differences between Pg. and CON mice

**FIGURE 4:**
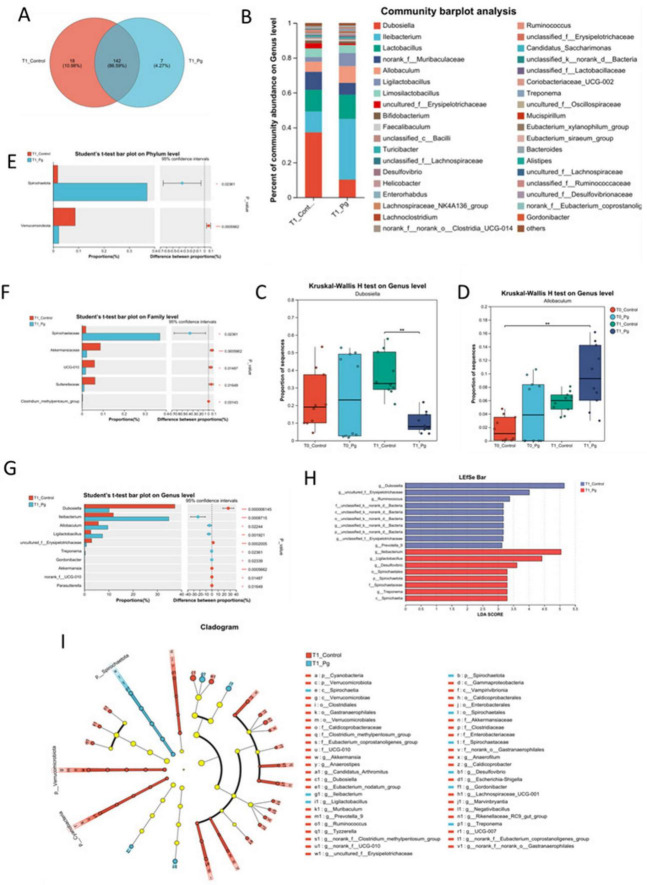
Gut species composition and difference analysis. **(A)** The Venn analysis for the species composition in the genus level. **(B)** Bar chart analysis of species composition at the genus level. **(C,D)** Multi-group test of difference at genus level. **(E–G)** Species difference test bar chart. The two-group difference tests for the CON and experimental groups were performed at the phylum level **(E)**, family level **(F)**, and genus level **(G)**, respectively. **(H)** LDA Score bar from the Lefse multilevel discriminant analysis of species differences. **(I)** Cladogram base on the Lefse multilevel discriminant analysis of species differences. *p < 0.05, **p < 0.01, ***p < 0.001.

The Venn diagram showed that there were 142 common genera between the CON and experimental groups, accounting for 86.59% of the total, while there were 18 unique genera in the CON group and 7 unique genera in the experimental group, accounting for 10.98% and 4.27%, respectively (Figure 4A). The Pg. group exhibited the presence of distinctive bacterial species, including g_norank_f_norank_o_SBR1031, whereas some other bacteria such as g_Prevotella_9 that presented in the CON group were undetected in the Pg. group. Meanwhile, the high abundance of genera shared by both groups included *g_Dubococcus* (23.74%), *g_Lactobacillus* (13.16%), and *g_Allobaculum* (7.71%) (Supplementary Figures S4A–D). And bar chart analysis of species composition at the genus level revealed significant differences in the species composition structure between the Pg. and Con groups. For example, *Dubosiella, norank_f_Muribaculaceae* and *uncultured_f_Erysipelotrichaceae* were significantly reduced in the Pg. group, while *Ileibacterium*, *Lactobacillus*, *Allobaculum*, and *Ligilactobacillus* were increased considerably (Figure 4B). Additionally, the discrepancy between the CON and the Pg. group samples is influenced by temporal changes. Multiple group difference tests were performed on the CON and Pg. group samples at both T0 and T1 time points, using Kruskal-Wallis as the statistical test. It was indicated that treatment with Pg. significantly altered the structure of the intestinal microflora in mice, for example, markedly lowering the proportion of *Dubosiella* (Figure 4C) but increasing the proportion of *Allobaculum* (Figure 4D). Next, the two-group difference tests for the CON and Pg. groups were performed at the phylum level (Figure 4E), family level (Figure 4F), and genus level (Figure 4G). The results showed a significant increase in the relative abundance of *Spirochaetota* and a significant decrease in *Verrucomicrobiota* in the Pg. group at the phylum level. At the family level, there was a significant increase in the proportion of *Spirochaetaceae* and a significant decrease in *Akkermansiaceae*, UCG-010 and *Sutterellaceae*, among others. At the genus level, there was a significant decrease in the percentage of *Dubosiella* and uncultured *f_Erysipelotrichaceae* in the Pg. group, and a significant increase in *Ileibacterium*, *Allobaculum*, and *Ligilactobacillus*. To further analyze the taxonomic hierarchical relationships from phylum to genus in the sample communities, Linear discriminant analysis Effect Size (LEfSe) evolutionary analysis was conducted, demonstrating notable discrepancies in specific microbial taxa (Figures 4H,I). Specifically, the Linear Discriminant Analysis (LDA) score bar graph illustrates that the three most prevalent keystone species in the CON group were identified as *Dubosiella*, uncultured *f_Erysipelotrichaceae*, and *Ruminococcis*. In contrast, the three most prevalent keystone species in the Pg. group were found to be *Ileibacterium*, *Ligilactobacillus*, and *Desulfovibrio* (Figure 4H).

#### 3.2.4 Prediction of the key functional pathways by the gut flora

**FIGURE 5:**
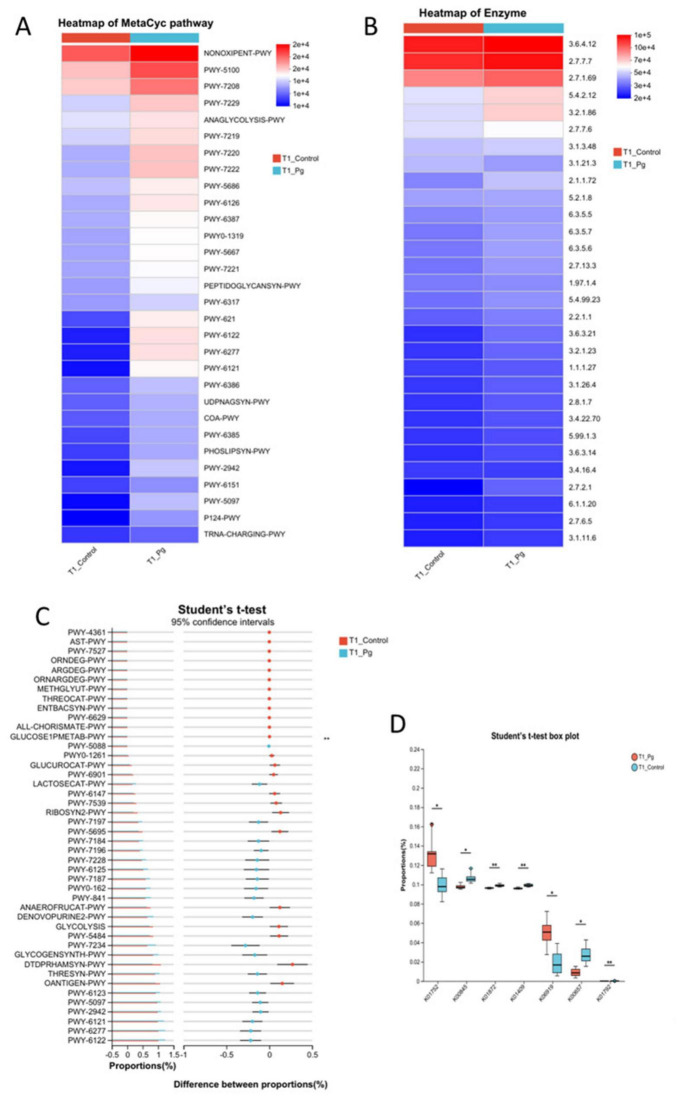
Functional predictive analytics. **(A)** Metabolic pathway analysis based on the Metacyc database using PICRUSt2. **(B)** Heatmap of the comparison analysis of the metabolic pathway based on the KEGG pathway tools on the enzyme level. **(C)** Histogram of confidence intervals for the test of differences between the T1 control and experimental groups, conducted relying on the MetaCyc database. **(D)** T1 *t*-test box plot between control and experimental groups according to KEGG database KO level. Different colors indicate different groups; significance markers are corrected *p*-values for the two-group test. *p ≤ 0.05, **p ≤ 0.001.

Further, we predicted the functions of bacterial microbiota using PICRUSt2 and the eggNOG database. Based on the metabolic pathway analysis using these databases, the results showed that the functions of bacteria were mainly involved in metabolism, with the highest abundance in amino acid metabolism (Figure 5A and Supplementary Figure S5A). Similarly, 16S rRNA sequencing data combined with KEGG functional predictions indicated that bacterial community functions were primarily associated with carbohydrate metabolism, as well as amino acid and nucleotide metabolic pathways (Figure 5B and Supplementary Figure S5B). By integrating more detailed KEGG data, the association of bacteria with the biosynthesis of amino acids, purine metabolism, pyrimidine metabolism, and pyruvate metabolism was further confirmed (Figures 5C,D and Supplementary Figure S5C).

### 3.3 Gastric administration of Pg. triggers marked changes in oral flora

#### 3.3.1 Changes in the species composition of the oral flora of mice after receiving *Pg*

**FIGURE 6:**
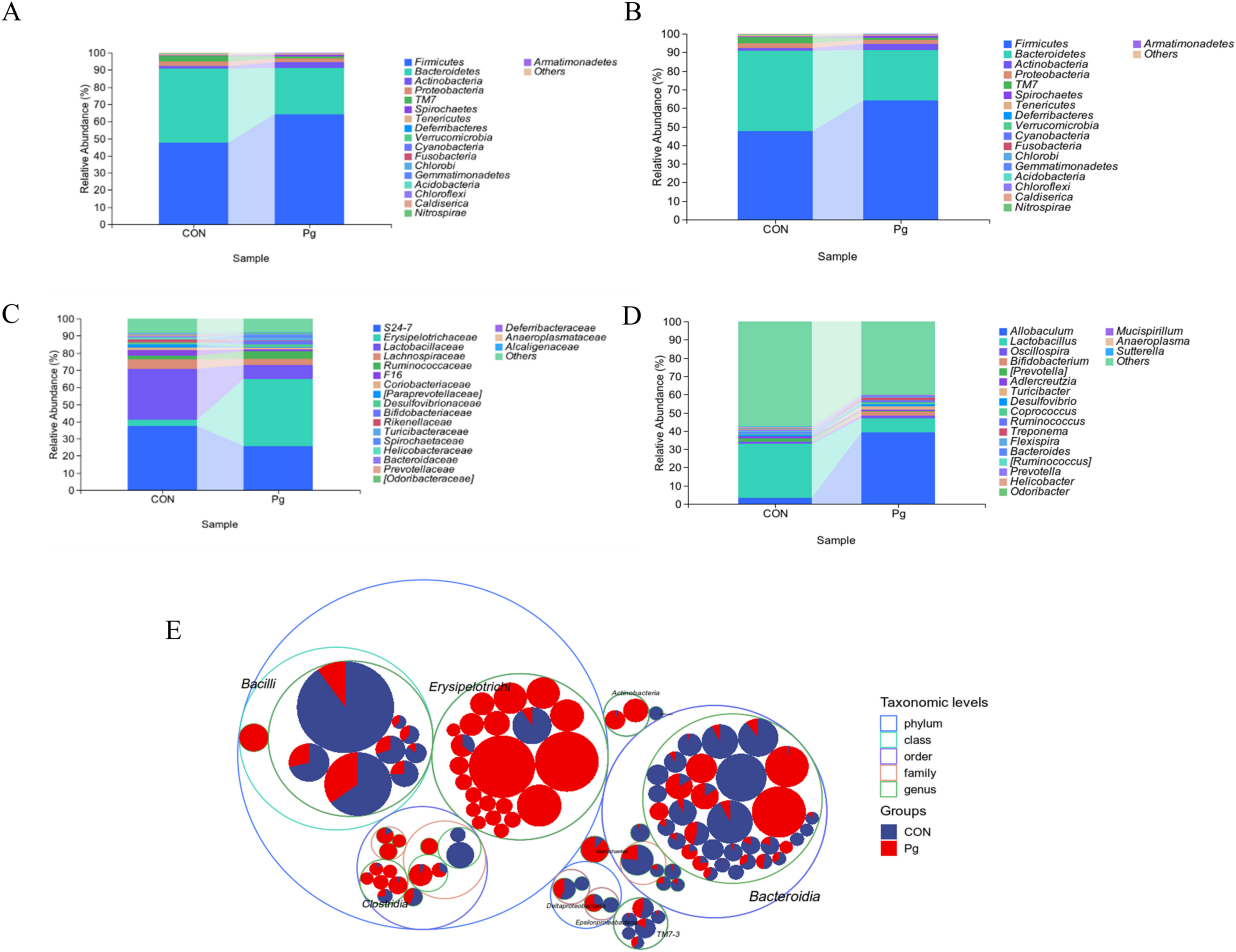
Analysis of the species composition of oral microorganisms. **(A)** Composition of phylum-level taxonomic abundance as exhibited by within-group means. **(B)** Composition of order level taxonomic abundance as exhibited by within-group means. **(C)** Composition of family-level taxonomic abundance exhibited by within-group means. **(D)** Composition of genus-level taxonomic abundance exhibited by within-group means. **(E)** Taxonomic Tree in Packed Circles.

The oral microbiota of mice was analyzed through sequencing, revealing significant differences between the CON and experimental Pg. groups. On average, 50,689.3 and 59,022.7 sequence pairs were obtained for the CON and *Pg.* groups, respectively (Supplementary Table S1A). Sequence lengths ranged from 246 to 443, with an average of 424 (Supplementary Table S1B). Taxonomic analysis using the *Greengenes* database showed notable differences in Amplicon Sequence Variants (ASVs) and taxonomic units between the groups (Supplementary Tables S2A,B and Supplementary Figure S6). At the phylum level, Firmicutes increased from 47.58% to 64.19%, while Bacteroidetes decreased from 43.35 to 27.11% in the Pg. group (Figure 6A). At the order level, *Erysipelotrichales* rose from 3.35 to 39.19%, whereas *Bacteroidetes* and *Lactobacillales* declined (Figure 6B). Family-level analysis showed increased *Erysipelotrichales* and *Turicibacter*, but decreased S24-7 and *Lactobacillaceae* in the Pg. group (Figure 6C). Genus-level changes included a rise in *Allobaculum* and *Turicibacter*, and a decline in Lactobacillus, *Prevotella*, and S24-7 (Figure 6D). ASV distribution further supported these findings, with *Allobaculum* significantly overexpressed and *Lactobacillus* underexpressed in the Pg. group (Figure 6E and Supplementary Figure S10). *Prevotella*, represented by ASV93, ASV21, and ASV98, showed no significant differences and was excluded as a key taxon. Consequently, *Allobaculum* and Lactobacillus were identified as primary factors for further analysis. Krona diagrams confirmed the substantial impact of Pg. treatment on oral microbiota composition (Supplementary Figures S7A–E).

#### 3.3.2 Analysis of oral species composition and differences between Pg. and CON mice

**FIGURE 7:**
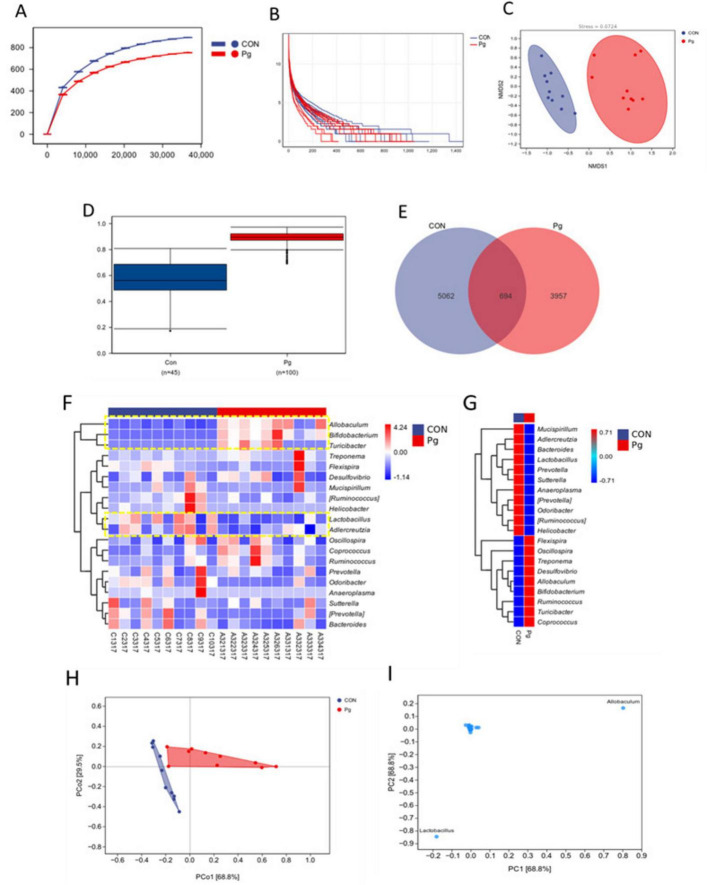
Oral species composition and difference analysis. **(A)** Rarefaction curve. **(B)** Rank abundance curve. **(C)** NMDS analysis for the oral flora. **(D)** Between-group differences analysis. **(E)** Venn analysis of the oral flora composition. **(F)** Heatmap of species composition at genus level. **(G)** Heatmap of genus-level species composition presented as mean values within groups. **(H,I)** PCA analysis for the oral flora composition.

Rarefaction curves plateaued with increasing sequencing depth, indicating sufficient data coverage for microbial diversity assessment. Pg. oral administration significantly reduced oral ASV counts, reflecting decreased microbial diversity (Figure 7A). Abundance rank curves showed greater species richness and more even distribution in the CON group, supported by Chao1 and Observed Species indices (Figure 7B). These results suggest that Pg. significantly alters murine oral microbiota composition and abundance (Figure 7C). Bray-Curtis analysis with adonis testing significant revealed intergroup differences (*R*^2^ = 0.38, *p* = 0.001) (Figure 7D). Venn analysis identified 5,062 CON-specific and 3,957 Pg-specific ASVs, with only 694 shared (Figure 7E). Heatmap visualization at the genus level confirmed distinct distribution patterns: *Allobaculum*, Bifidobacterium, and Turicibacter were enriched in the Pg. group, while Lactobacillus and *Adlercreutzia* dominated in the CON group (Figures 7F,G). PCA demonstrated clear group separation, with principal components explaining 68.8% and 29.5% of variance, respectively (Figure 7H). Variable loading analysis identified *Allobaculum* and *Lactobacillus* as key drivers of community structure differences (Figure 7I).

**FIGURE 8:**
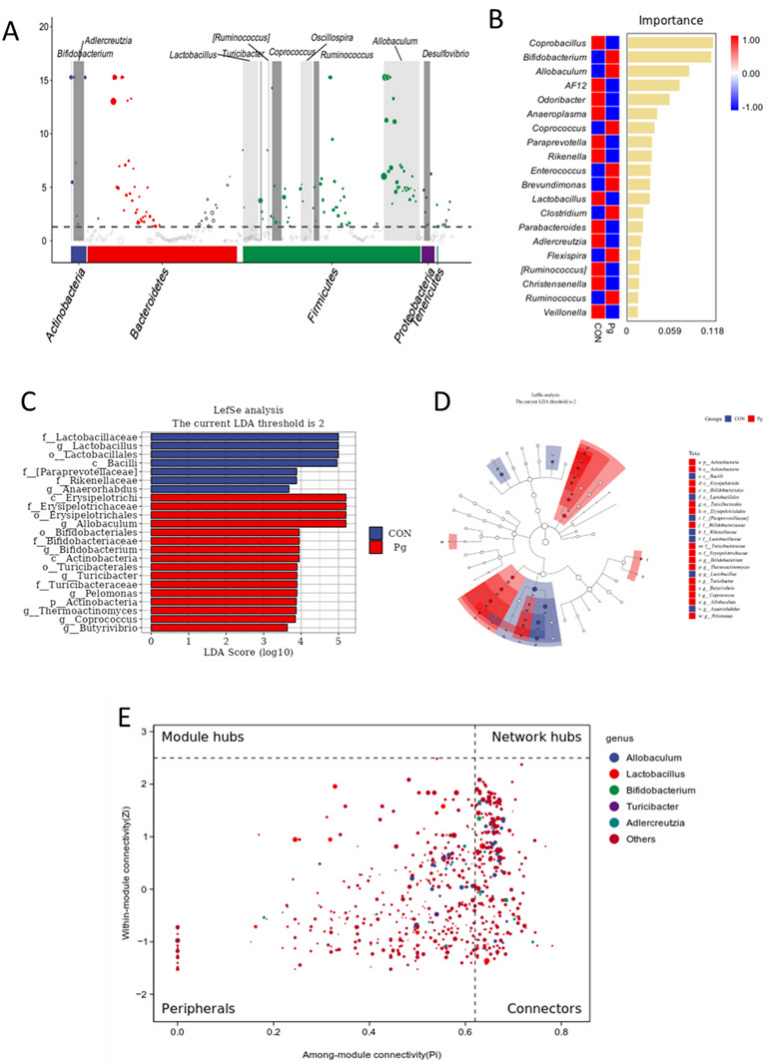
Analysis of species differences and keystone species. **(A)** MetagenomeSeq analysis. The threshold of ASV is 0.0002. **(B)** Random forest analysis. **(C)** LDA score. Illustrated in the figure are species with LDA scores greater than 3.6. **(D)** Linear discriminant analysis Effect Size (LEfSe) analysis. **(E)** ZiPi co-occurrence plot analysis.

To identify key species driving differences in microflora structure between the CON and Pg. groups, we conducted MetagenomeSeq, Random Forest, and LEfSe analyses. MetagenomeSeq analysis revealed that Allobaculum and Lactobacillus were the most abundant and significantly different ASVs (threshold: 0.0002), consistent with species composition heatmaps and taxonomic abundance analyses (Figure 8A). Random Forest analysis identified *Coprobacillus*, *Bifidobacterium*, *Allobaculum*, and Lactobacillus as potential marker species for group differences (Figure 8B). The top keystone species in the CON group were *Bacilli*, *Lactobacillales*, *Lactobacillaceae* and Lactobacillus, while in the Pg. group, they were *Erysipelotrichi*, *Erysipelotrichales, Erysipelotrichaceae*, and *Allobaculum* (Figure 8C). These species belong to the same evolutionary lineage. Pg. oral administration induced significant changes in oral microflora, with *Allobaculum* and *Lactobacillus* as the primary differential markers (Figure 8D). Intra-module (Zi) and inter-module (Pi) connectivity analyses further confirmed *Allobaculum* and *Bifidobacterium* as keystone species, highlighting their importance in microbial networks (Figure 8E).

#### 3.3.3 Prediction of the potential functional pathways by the oral flora

**FIGURE 9:**
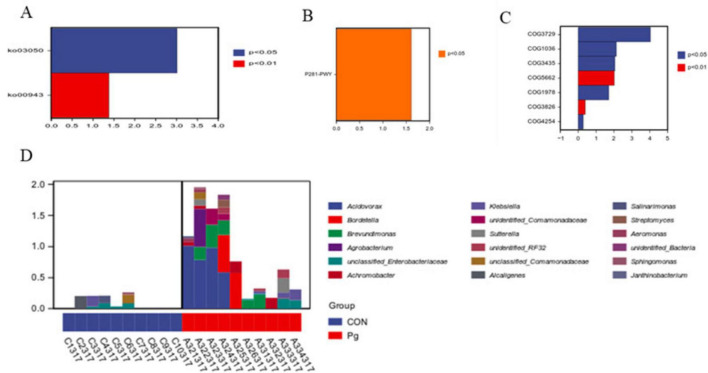
Functional potential prediction. **(A)** Differential analysis of metabolic pathways based on KEGG pathway. **(B)** Differential analysis of metabolic pathways based on MetaCyc database. **(C)** COG-based differential analysis of metabolic pathways. **(D)** Species composition map analysis based on the P281-PWY metabolic pathway.

The KEGG pathway analysis showed pathways with absolute LogFC values greater than 1 and *p*-values and adj *P* < 0.05 for KO03050 and KO00943 (Figure 9A), respectively. Similarly, MetaCyc analysis highlighted P281-PWY as a key altered pathway under the same thresholds (Figure 9B). COG analysis further revealed differentially represented functional categories, including COG3729, COG1036, COG3435, COG5662, and COG1978 (Figure 9C). Species composition analysis of the metabolic pathways revealed that among the identified species, Acidovorax ranked highest, indicating its predominant contribution to the P281-PWY pathway (Figure 9D). Subsequently, following a statistical analysis of metabolic pathways based on KEGG (Supplementary Figures S8A,B), MetaCyc (Supplementary Figures S8C,D), and COG (Supplementary Figures S8E,F), it was determined that a more pronounced disparity was evident between the two groups at the PCoA2 level.

Serum analysis of ASD model mice showed that the levels of inflammatory factors (IL-6 and IL-1β) were significantly increased (Supplementary Figure S1A,B), and the levels of short-chain fatty acids (SCFA) were significantly decreased in the experimental group compared with the control group (Supplementary Figure S9). Notably, the impaired degradation pathway results in accumulation of 3-phenylpropionate, a metabolic intermediate that cannot be further catabolized into non-toxic SCFAs. In animal models, such metabolic disturbances indicate gut microbial dysbiosis and a potential mechanistic link to the development of asd symptomatic behaviors.

#### 3.3.4 The comprehensive analysis of the relationship between behavioral changes and the enriched taxa, as well as the link between Pg-altered microbiota in oral and gastrointestinal environments

**FIGURE 10:**
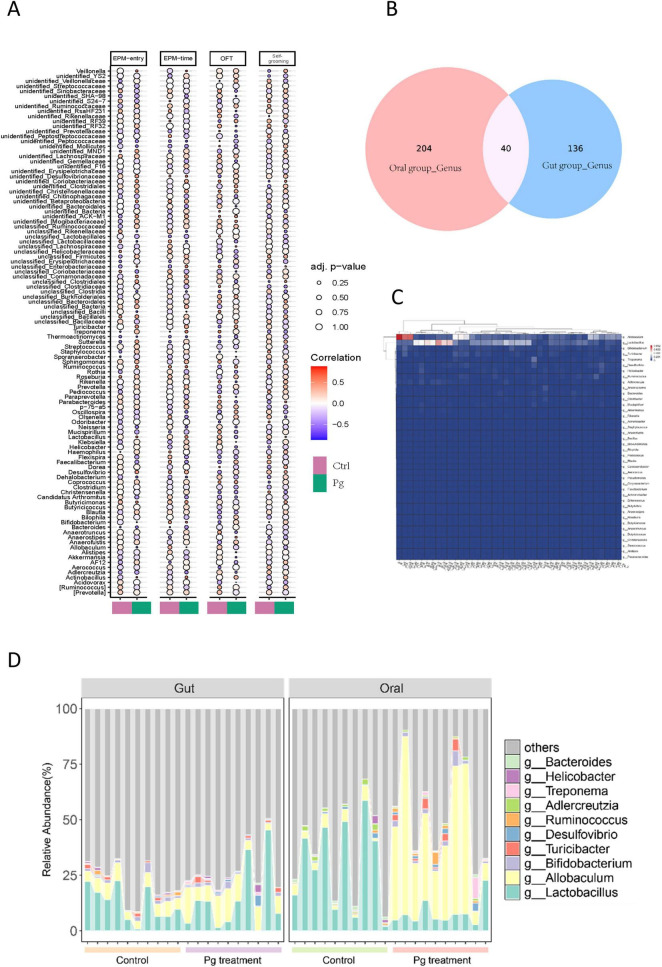
The relationship between behavioral changes and regulated taxa, alongside the link between Pg-altered microbiota in oral and gastrointestinal environments. **(A)** Correlation analysis between Gut microbiota abundance and behavioral alterations. **(B)** Venn Diagram Analysis of gut microbiota and oral microbiota. **(C)** Hierarchical clustering analysis of core microbiota relative abundance profiles. **(D)** Stacked bar chart illustrating relative abundance of both gut and oral core genera.

Combining the results of animal behavioral experiments with the analysis of relevant data, revealed a robust correlation between Pg. induced gut microbiota alterations and significant behavioral modifications in mice following oral Pg. administration (Figure 10A). For example, analysis of the experimental data revealed that *Klebsiella*, *Sporanaerobacter*, and *Alistipes* exhibited a significant positive correlation with open field test in mice under the influence of oral *Pg*. In contrast, *Ruminococcus*, *Clostridiaceae*, and *Anaerofustis* demonstrated a pronounced negative correlation with open field test outcomes under the same experimental conditions. A positive correlation is evident between *Firmicutes* and the elevated plus maze experiments, while *Butyricicoccus shows the opposite trend*. Besides, *Bacilli* inhibits self-grooming of mice (Figure 10A).

To investigate the role of Pg. within a specific microbial community through both the gut and the oral microbiota alterations, we conducted an analysis of all genera identified in both niches. The Veen diagram revealed that 40 genera were present in both locations after Pg. treatment (Figure 10B). Further, a clustering analysis focusing on these 40 genera based on their relative abundance was conducted. The heatmap showed that only 14 of these genera exhibited significant changes in relative abundance, among which Allobaculum and Lactobacillus were notable, displaying significant differences between the oral cavity and gut perturbations by Pg. treatment (Figure 10C). Specifically, Allobaculum in the oral cavity showed the most significant changes in relative abundance in response to Pg (Figure 10C). Next, to visually compare these co-existing genera in both situations, which demonstrated relatively high abundance and sensitivity to Pg. treatment, a stacked bar chart illustrating their relative abundance was generated. The data indicated that *Allobaculum*’s abundance increased in both the oral cavity and gut after Pg. treatment, while Lactobacillus showed a decrease in abundance, particularly pronounced in the oral cavity (Figure 10D).

## 4 Discussion

People with ASD usually exhibit complex behavioral conditions, including significant social difficulties and repetitive behaviors. Various genetic variations and environmental risks associate with ASD. Studies have shown that the composition of the gut microbiota in ASD patients is different from healthy people, and these differences may be related to the development of ASD symptoms. For example, in the gut microbiota of ASD children, the relative abundance of certain bacterial genera increases, such as *Bacteroides*, *Parabacteroides*, and *Clostridium*, while the abundance of some beneficial bacteria, such as *Coprococcus* and *Bifidobacterium* decreases (Chen et al., 2024). In addition, by fecal microbiota transplantation, the gut microbiota of ASD patients can be transplanted into germ-free mice, which can induce ASD-like behaviors, further indicating the potential link between the gut microbiota and ASD behaviors (Wang et al., 2023).

In the phenotypic exploration phase, we conducted behavioral tests on mice with oral bacteria transplantation and found that Pg. transplantation could promote the occurrence of ASD symptoms in mice, with social impairment being a core symptom of ASD. The above results comprehensively reflect the social behavior deficits in *Pg*-transplanted mice. However, there are currently few studies on the relationship between Pg. and ASD, and most of the research on Pg. in non-oral diseases focuses on AD, which has been found to cause cognitive function decline similar to AD after infection in mice (Ma et al., 2023). In addition, several studies have consistently shown that the transplantation of gut microbiota from ASD donors and co-housing with ASD mice can cause social deficits in mice.

In the mechanistic investigation phase, we found that gut *Dubosiella* and *Erysipelotrichaceae* were both decreased in the T1 Pg. group. Notably, *Dubosiella* is a genus of the family *Erysipelotrichaceae*, and its two type species such as *Dubosiella newyorkensis* have been well validated to be intestinal probiotics which can modulate the immune response through the production of short-chain fatty acids and L-lysine (Zhang et al., 2024), enhance the function of vascular endothelial cells, reduce oxidative stress, and alter the gut flora structure (Liu et al., 2023). Moreover, *Erysipelotrichales* were found to be significantly more prevalent in the oral cavity of the Pg. group, suggesting that Pg. may be responsible for migrating certain key bacteria from the intestine into the mouth cavity. Therefore, the *Pg-mediated* unhealthy status of the gut flora and even the ASD like behavior may be partially attributed to this suppressed probiotic in the gut. However, the more detailed mechanisms still remain to be revealed by future studies.

Numerous studies have been done on the connection between ASD and flora, and thorough comparisons between these findings aid in presenting a comprehensive grasp of its intricate role. As to one of the relevant studies, significant alterations in Bacteroidetes and Firmicutes have been identified in conjunction with the autistic-like social behaviors in the offspring mice, which is consistent with our findings that *Pg*-treated mice with ASD-like behaviors have marked differences regarding saliva Bacteroidetes and Firmicutes. According to another research, children with ASD had a less diversified gut microbiome with greater amounts of *Lactobacillus*, *Bacteroidetes*, Sarcina, and other bacteria and lower levels of *Bifidobacterium* and *Firmicutes* (Li et al., 2017). However, in our study, it was found that the *Pg*-treated mice exhibited a lower level of *Lactobacillus* in the gut, but a higher level of *Lactobacillus* in oral microbiota. Based on the fact that *Lactobacillus* is one of the genera *Firmicutes*, the comprehensive understanding of the above-mentioned perspective may need more logical thinkings. We inferred that there may be much deeper unknown unidentified mechanisms underlying this inconsistence.

Collectively, this study confirmed that long-term Pg. administration inhibits the ASD development. Targeting this bacterium should be beneficial to improve the ASD sufferers and promising for clinical use. The mechanism for this phenomenon may be related to *Pg*’s role in the regulation of the re-distribution of key taxon between the gut and oral microcosm, such as the *Dubosiella* and *Lactobacillus*. However, further in-depth research and thorough analysis are still required to identify the precise biochemical mechanism or core that underlies *Pg*’s significant effects on the development of ASD.

## 5 Conclusion

In this study, we sought to assess the impact of gingival pustulosis on the ASD phenotype, whilst also exploring its principal mechanisms. To this end, we conducted a comparative analysis of the behavioral performance of gingival pustulosis-treated mice and their normal counterparts. Furthermore, 16S rRNA sequencing analysis of the oral and intestinal flora found significant differences in the compositional structure and diversity of the flora in the CON and Pg. groups, suggesting Pg, its key taxa, and possible functional downstream metabolites were responsible for the exacerbation of ASD.

## Data Availability

The original contributions presented in the study are publicly available. This data can be found in here: https://www.ncbi.nlm.nih.gov/, accession numbers: SRR34732525-SRR34732534.
