# Supplementary material for: Porphyromonas gingivalis deteriorates autism spectrum disorders by disturbing the gut and oral microbiota
Source: Front Microbiol. 2025 Aug 6;16:1579128. doi: 10.3389/fmicb.2025.1579128 (PMC12364871; doi:10.3389/fmicb.2025.1579128)
Supplement: Supplementary file 1 [file Data_Sheet_1.pdf]

Supporting Information

The levels of proinflammatory factors increased in mice

In the present study, ELISA quantification showed that serum proinflammatory cytokines were significantly increased after Pg infection in mice, which suggested the ability of Pg to induce systemic inflammation in mice, highlighting the ability of Pg to cause systemic inflammation. In addition, related behavioral experiments also proved the effect of Pg on the behavior of mice. Detailed data can be found in the manuscript site and Figure S1 C-D.

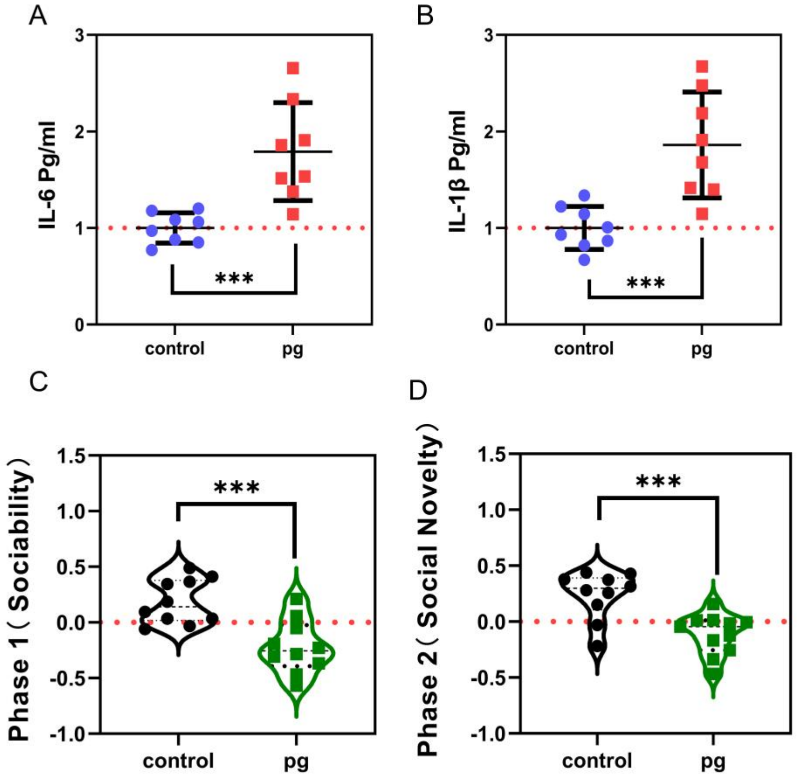

Figure S1 A: Results of IL-6 level testing. B: Results of IL-1β level testing.C-D:Social preference index of the hatchback experiment in mice

Analysis of the α diversity of the intestinal flora of experimental mice

A total of 50 samples were tested using 16S rRNA high-throughput sequencing, divided into two time points (T0 and T1) with 10 samples in the control group and 15 samples in the experimental group. The statistical table of sequencing information can be seen in Figure S2 A. The amplified region of the sequence was 338F_806R, with an insert length of 468 bp. PE250 double-end sequencing was employed, yielding

651,914 original sequences and 1,629,786,000 bp in the optimized sequence. Following quality control splicing of double-ended sequences, the number of bases was 1629786000, the number of optimised sequences was 3259572, and the number of optimised bases was 1389087323. As illustrated in Figure S2 B, the length of the optimised sequence is predominantly distributed within the interval of 421-440, with a minimum length of 200 bp, a maximum length of 452 bp, and an average length of 426 bp. This demonstrates that the data obtained from the sequencing are reasonably distributed. Figure S2 C and Figure S2 D are sample Bray Curtis distance heatmap, the figure with X-axis, Y-axis identifies the samples, the distance between the samples in different colours, the colour shown in the legend on the top right represents the value of the distance between the two samples, which can be seen between the groups of samples. The sample-level clustering demonstrates the grouping of samples within each group. The results indicate the presence of discernible characteristics with regard to the clustering patterns observed in the four groups. Notably, the two T0 groups exhibit a cross fertilised clustering, whereas the two T1 groups display a distinct clustering pattern. The results reflect both the significant effect of different treatments on the β-diversity of the intestinal flora at the same time point and the fact that the intestinal flora was also somewhat altered between the two time points.

A

Raw Sequence Information	Amplified Region	Insert size(bp)	Sequencing Length	Raw Reads	Total Base(bp)
	338F_806R	468	PE250	3259572	814893000
Optimized Sequence Information	Amplified Region	Samples	Sequences	Bases(bp)	Average Length
	338F_806R	50	3259572	1389087323	426

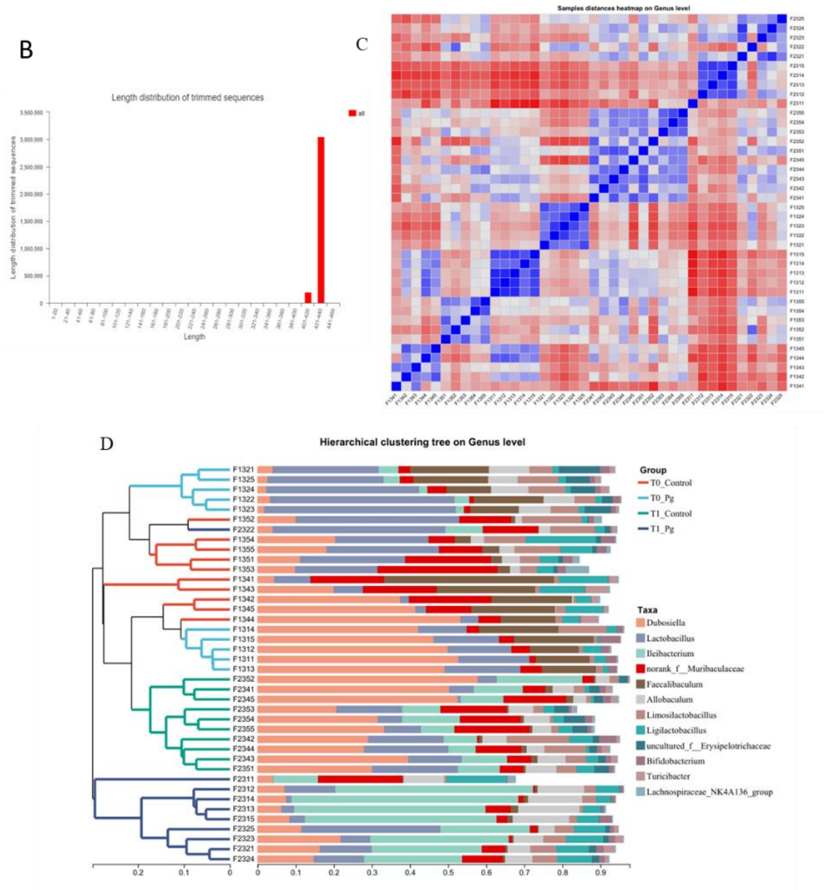

Figure S2 A: Statistical table of 16S rRNA sequencing information. B: Statistical map of sequence length distribution after optimisation. C: Heat map of Bray Curtis distances of samples at genus level. D: Hierarchical clustering analysis of samples at genus level.

Subsequently, as shown in Figure S3 A-F, the indices corresponding to the individual samples of ace, chao, Shannon, Simpson, sobs, and coverages calculated at the OTU level were analysed and for the statistical analyses were performed for simpson and Shannon. (Figure S3 G-H) . The results showed that *Pg* administration reduced the ace, chao, Shannon, Simpson, and sobs indices, which responded to the fact that the treatment factor had a significant tendency to reduce the ecological richness, diversity, and homogeneity of the gut microbes in mice, and the difference was statistically significant. Figure S3 I illustrates that at the NMDS1 level, the T1 experimental group exhibited notable differences from the remaining three groups. There was a considerable degree of overlap in sample distribution between the two T0

groups, and a notable overlap between the T1 control group and the former two. These findings once again indicate that the T1 experimental treatment resulted in alterations to the beta diversity of the mouse flora.

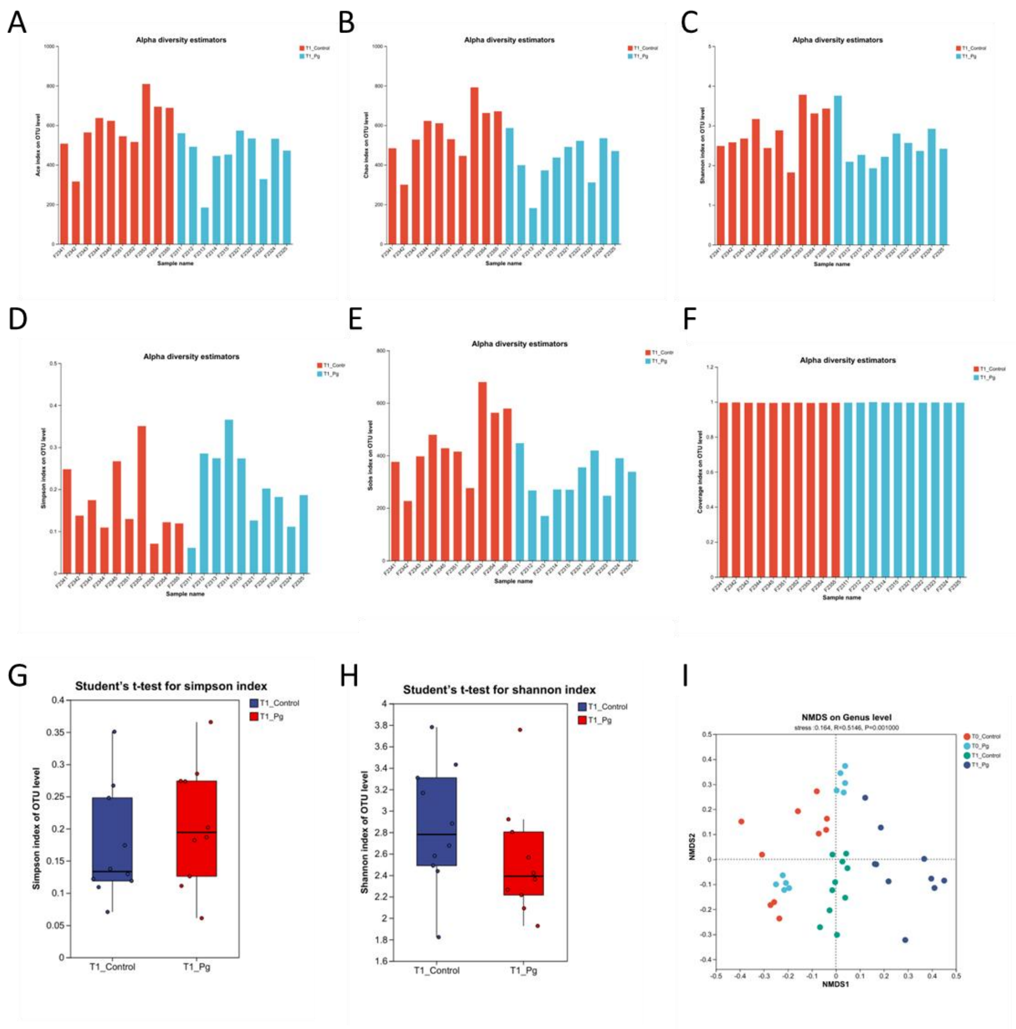

Fig S3 A-F: The values of each index corresponding to each sample obtained from Alpha diversity analysis. G-H: A comparative analysis of the between-group indices proposed by simpson and shannon. I. NMDS analysis of the beta diversity of the gut microbiota

Intestinal species composition and differences between Pg and CON mice

As shown in Figure S4 A-C, the bacterial species of the CON group and the *Pg* group can be obtained through the analysis of the assay, in which the *Pg* group not only exhibits unique bacterial species, but also has bacterial species common to the CON group. Figure S4 D depicts a heat map of community composition, wherein the horizontal axis represents the sample name and the vertical axis denotes the species name. The colour gradient of each block illustrates the abundance variation of distinct

species within each sample at the genus level. The colour of the top bar serves to represent the grouping.

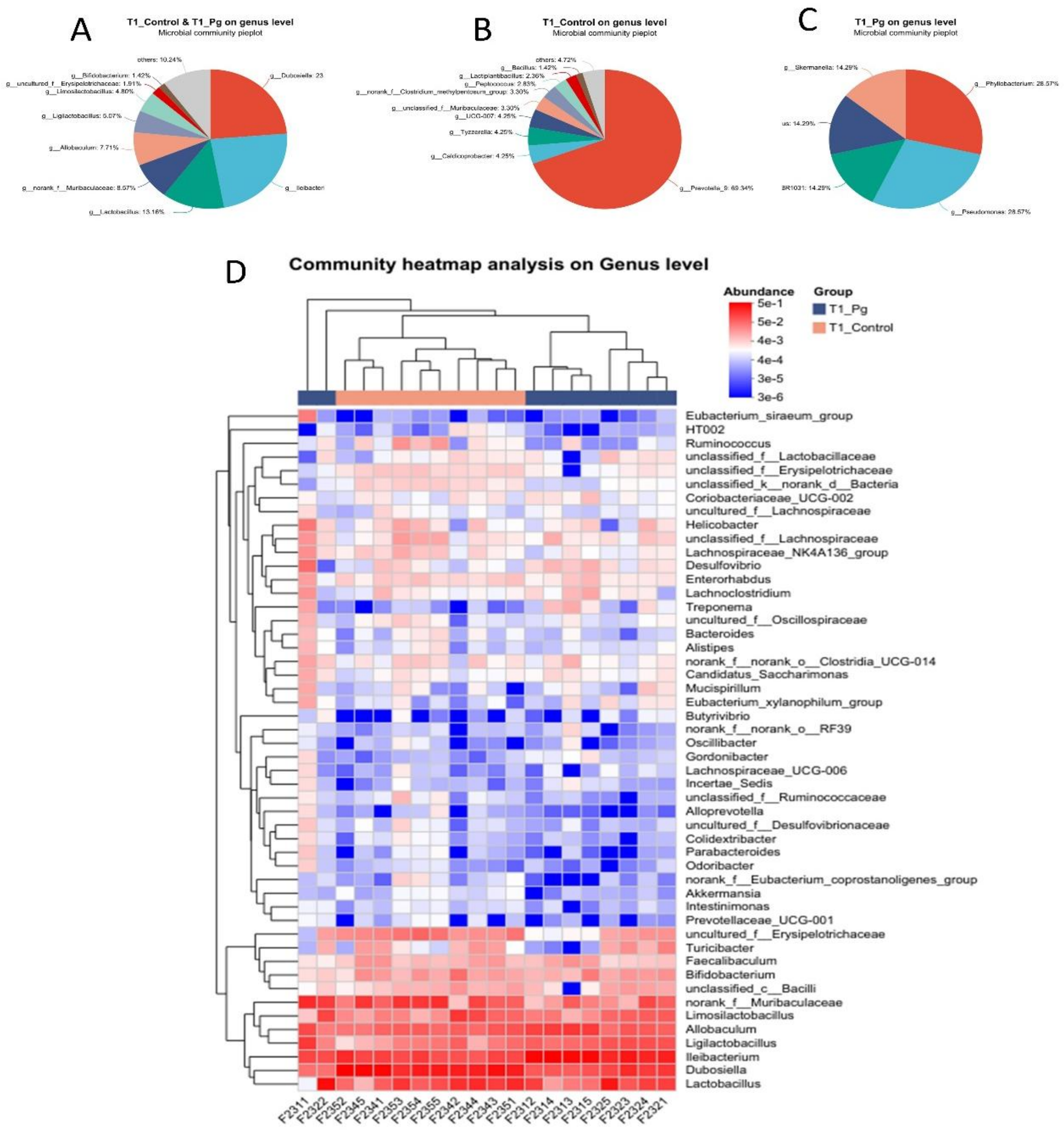

Figure S4 A-C: Species composition pie charts for each treatment group. D: Heat map of community composition

Prediction of the key functional pathways by the gut flora

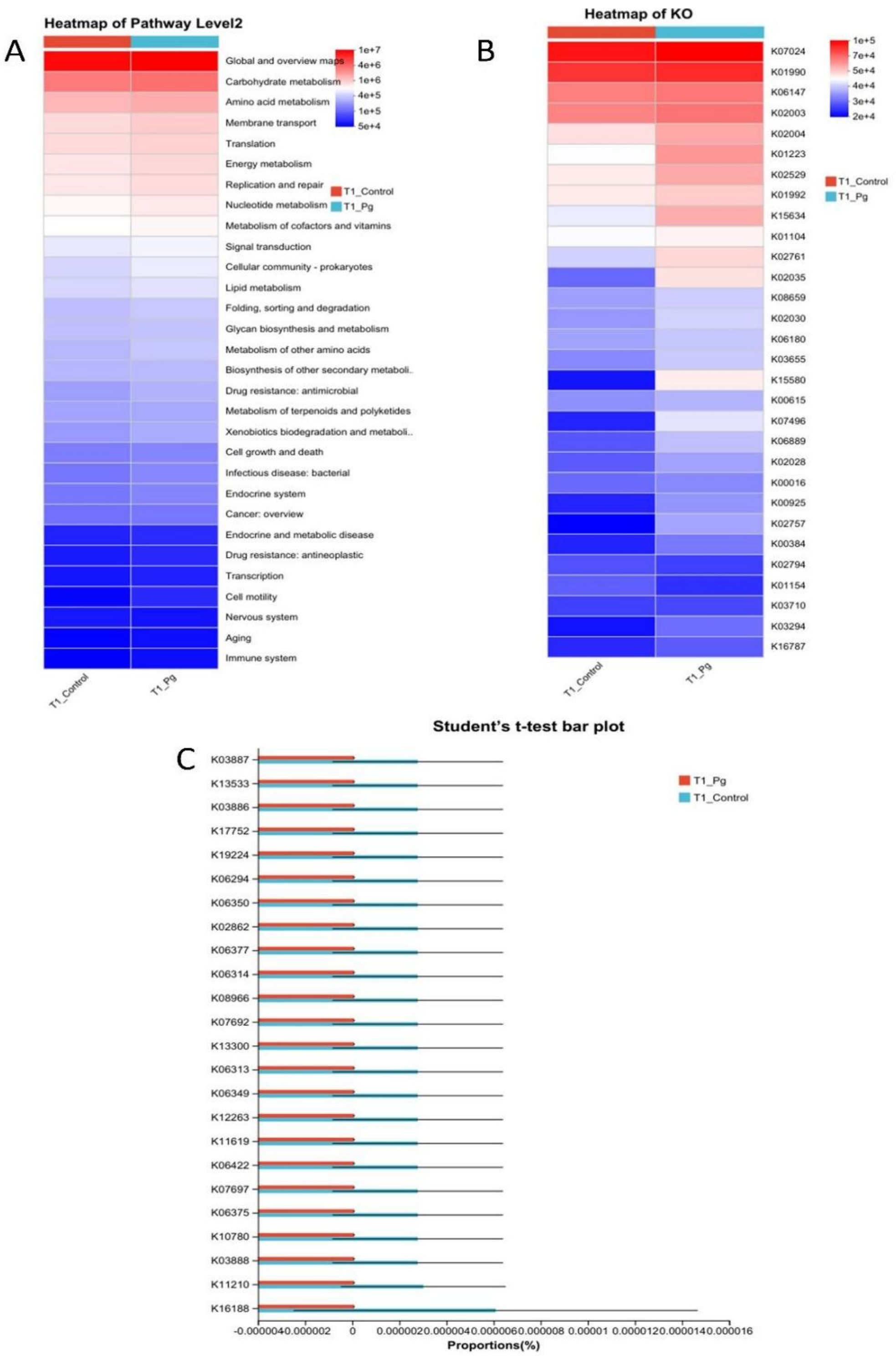

Figure S5. A-B: Metabolic pathway analysis based on the KEGG database using PICRUSt2. at the 2nd level (A) and KO level (B). C: Bar chart of T-test between T1 control group and experimental group based on KO level in KEGG database. (The pathways listed here all meet the following conditions that the P-value is less than 0.05 and the FDR is >30 for KO entries. The lengths of the bands on the abscissa represent the relative average abundance of a given KO in different subgroups.)

Analysis of the species composition of the oral flora of experimental mice

Table S1 A show that the statistical table of sequencing volume of each sample shows that the control group obtained an average of 101558.1 pairs of original sequences, while the experimental group obtained an average of 107005.9 pairs of original sequences per sample; after preliminary quality filtering, the control group obtained 95867.8 pairs of sequences, while the experimental group obtained an average of 101496.6 pairs of sequences per sample; after denoising, the control group obtained an average of 93725 sequences, and the experimental group obtained an average of 99347 sequences; after splicing, the control group obtained an average of 81942.3 pairs of sequences, and the experimental group obtained an average of 99347 pairs of sequences; and after splicing, the control group obtained an average of 81942.3 pairs of sequences. After denoising, the control group obtained an average of 93,725 sequences, while the experimental group obtained an average of 99,347 sequences; after splicing, the control group obtained an average of 81,942.3 pairs of sequences, while the experimental group obtained an average of 88,334.2 pairs of sequences; and after removing chimeras, the control group obtained an average of 50,689.3 pairs of sequences, while the experimental group obtained an average of 59,022.7 pairs of sequences(a total of 20 samples, 10 each in the control and experimental groups). It can be seen that the average values of sequences per sample measured in the experimental group were higher than those in the control group, suggesting that *Porphyromonas gingivalis* treatment has a certain effect on the abundance of oral biota in animals. Subsequently, we performed a draw leveling of the ASV abundance scale (with the depth set to 95% of the minimum sample sequence volume). Table S1 A show that the analysis of the sequence length distribution shows that the sequence length distribution ranges from 246 to 443, and the average sequence length is 424.

A

SampleID	Input	Filtered	Denoised	Merged	Non-chimeric	Non-singleton
C1317	90177	85112	83427	73870	38712	38502
C2317	97750	91636	89690	78308	48842	48656
C3317	108210	102524	100764	92254	64598	64507
C4317	93190	88072	85892	71989	39064	38836
C5317	105730	99327	97926	91009	63001	62941
C6317	102389	95934	93477	79164	44149	43892
C7317	107664	101584	99911	92315	51910	51821
C8317	104245	99353	95943	79263	51129	50953
C9317	102830	97218	93677	71348	41430	41047
C10317	103396	97918	96543	89903	64058	64012
Average	101558.1	95867.8	93725	81942.3	50689.3	50516.7
A321317	104324	98612	96435	85464	61219	61156
A322317	117921	111925	109130	93027	60525	60395
A323317	110266	105346	103243	93629	59450	59340
A324317	113900	107373	104348	87489	54561	54410
A325317	112672	107270	103949	86510	57119	56918
A326317	113812	107988	106643	100792	67311	67280
A331317	98907	94643	93336	87762	63860	63796
A332317	111598	104592	102482	91572	60185	60054
A333317	90232	85331	83088	70799	43695	43501
A334317	96427	91886	90816	86298	62302	62279
Average	107005.9	101496.6	99347	88334.2	59022.7	58912.9

B

length	count	length*count
246	2	492
261	2	522
262	2	524
271	2	542
273	3	819
278	3	834
284	3	852
293	2	586
301	2	602
304	2	608
305	4	1220
311	4	1244
314	2	628
318	10	3180
319	3	957
320	3	960
321	2	642
326	29	9454
330	2	660
333	2	666
334	2	668
336	11	3696
337	2	674
338	21	7098
339	20	6780
340	5	1700
343	2	686
348	4	1392
351	2	702
354	2	708
357	2	714
358	5	1790
362	10	3620
363	2	726
365	6	2190
366	5	1830
367	4	1468
368	10	3680
369	9	3321
370	7	2590
371	6	2226
374	5	1870
376	5	1880
378	9	3402
379	2	758
380	5	1900
384	5	1920
385	5	1925
386	13	5018
387	3	1161
389	7	2723
391	5	1955
392	4	1568
393	4	1572
402	490	196980
403	6	2418
404	8988	3631152
405	74292	30088260
406	34324	13935544
407	37892	15422044
408	34213	13958904
409	421	172189
410	88	36080
411	2517	1034487
412	3877	1597324
413	21	8673
414	3	1242
415	2	830
416	2	832
417	83	34611
419	13	5447
421	40	16840
422	794	335068
423	25074	10606302
424	203176	86146624
425	143206	60862550
426	1125	479250
427	9037	3858799
428	146	62488
429	206883	88752807
430	309323	133008890
431	735	316785
432	39	16848
433	7	3031
438	3	1314
443	7	3101
Sum	1097120	464699617
Everage		423.5631626

Table S1 A: Sequencing volume statistics of every sample. B: Table of sequences lengths distribution

Table S2 A illustrates that by comparing and scoring the measured sequence information with the Greengenes species taxonomic annotation reference database, it can be determined that the mean number of ASVs obtained in the control group was 896.2, while the mean number of ASVs obtained in the experimental group was 754. The data in Table S2 B and Figure S6 , on the other hand, show that, as far as the mean number of taxa is concerned, one domain was identified for each treatment group in this experiment. At the phylum level, the control group averaged 10.9

compared to 10.5 in the experimental group; at the order level, the control group averaged 18.6 compared to 17.7; at the order level, the control group averaged 24.6 compared to 24. 9; at the family level, the control group averaged 35.9 compared to 38.2; at the genus level, the control group averaged 42.5 compared to 42.7; at the species level, the control group averaged 19.8 compared to 19.1 for the experimental group.

A

ID	domain	phylum	class	order	family	genus	species	unclassified	Sum of ASV
C1317	12	1	2	73	602	134	30	0	854
C2317	14	3	0	151	544	164	28	0	904
C3317	17	1	3	110	442	139	34	0	746
C4317	20	1	1	200	651	147	28	0	1048
C5317	9	3	1	56	393	107	41	0	610
C6317	29	1	3	198	753	155	37	0	1176
C7317	24	2	2	81	350	124	36	0	619
C8317	22	6	1	231	395	250	74	0	979
C9317	34	0	3	385	781	209	47	0	1459
C10317	7	1	0	97	322	114	26	0	567
Average									896.2
A321317	20	7	0	189	305	178	34	0	733
A322317	32	4	1	194	533	255	35	0	1054
A323317	27	12	3	114	281	180	47	0	664
A324317	36	3	3	195	432	249	46	0	964
A325317	28	5	4	295	427	232	36	0	1027
A326317	17	1	1	87	144	145	23	0	418
A331317	26	6	1	75	192	92	23	0	415
A332317	10	3	2	113	547	168	44	0	887
A333317	27	8	1	179	570	204	38	0	1027
A334317	17	4	0	71	128	110	21	0	351
Average									754

B

ID	domain	phylum	class	order	family	genus	species
C1317	1	9	16	24	33	41	19
C2317	1	10	18	24	37	41	21
C3317	1	14	26	33	42	43	21
C4317	1	11	19	24	34	45	20
C5317	1	12	21	27	38	42	20
C6317	1	11	18	26	37	46	21
C7317	1	11	18	21	35	36	16
C8317	1	12	18	24	38	45	22
C9317	1	11	17	21	30	42	21
C10317	1	8	15	22	35	44	17
Average	1	10.9	18.6	24.6	35.9	42.5	19.8
A321317	1	11	17	25	42	44	19
A322317	1	12	19	27	39	48	20
A323317	1	12	21	27	44	46	20
A324317	1	12	20	28	43	50	22
A325317	1	10	17	26	37	42	22
A326317	1	9	16	24	39	41	15
A331317	1	8	14	19	27	33	18
A332317	1	11	19	28	40	46	19
A333317	1	11	19	27	40	44	21
A334317	1	9	15	18	31	33	15
Average	1	10.5	17.7	24.9	38.2	42.7	19.1

Table S1 A: Annotated list of species classification. B: Statistical data table for the number of categorical units in each sample

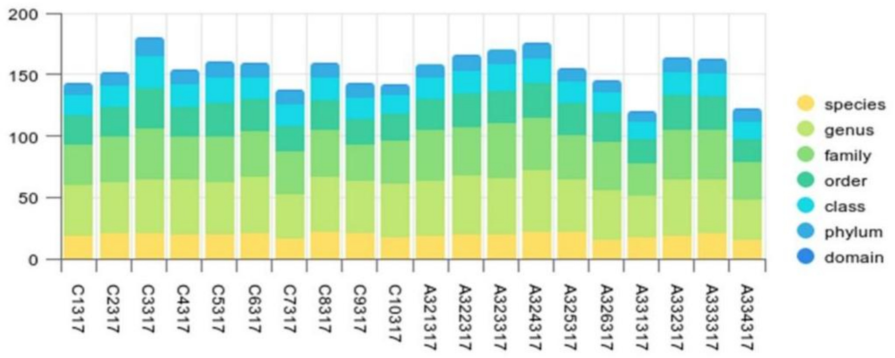

Figure S6. Statistical chart of the number of categorical units in each sample

Changes in the species composition of the oral flora of mice after receiving Pg

Figure S7 shows the analysis of taxonomic composition at the phylum (A), family (B) and genus (C) levels. Through Krona's species composition diagram (Figure S7 D-E), it was likewise found that Pg treatment exerted a great influence on the composition of the mice's oral flora.

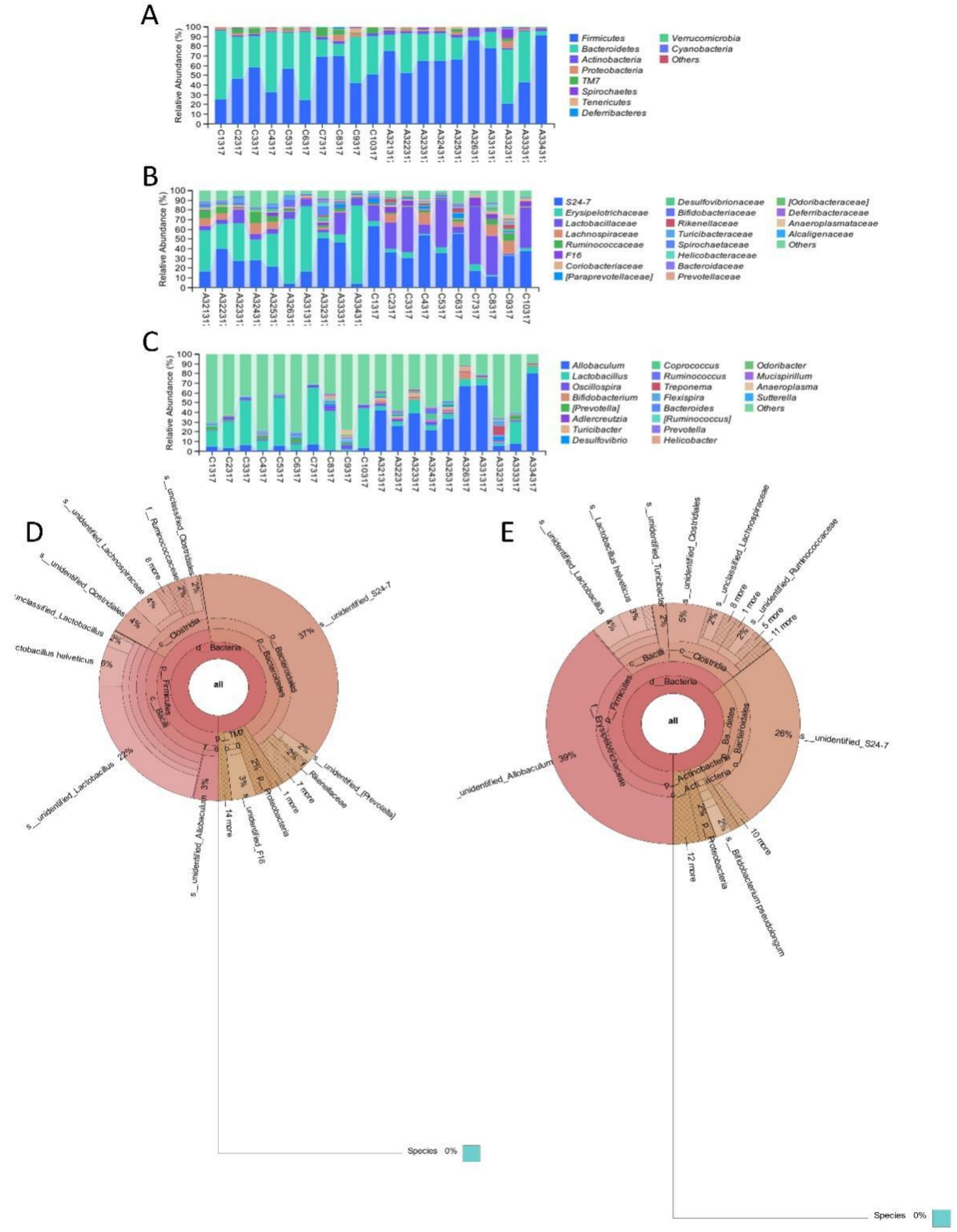

Figure S7 A-C: Percentage stacked bar chart for the taxonomic analysis. Taxonomic composition analysis at the phylum (A), family (B) and the genus (C) level. D-E: Krona species composition analysis. D is for the Con group, and E is for the *Pg* group.

Screening of key pathways in the oral flora of experimental mice

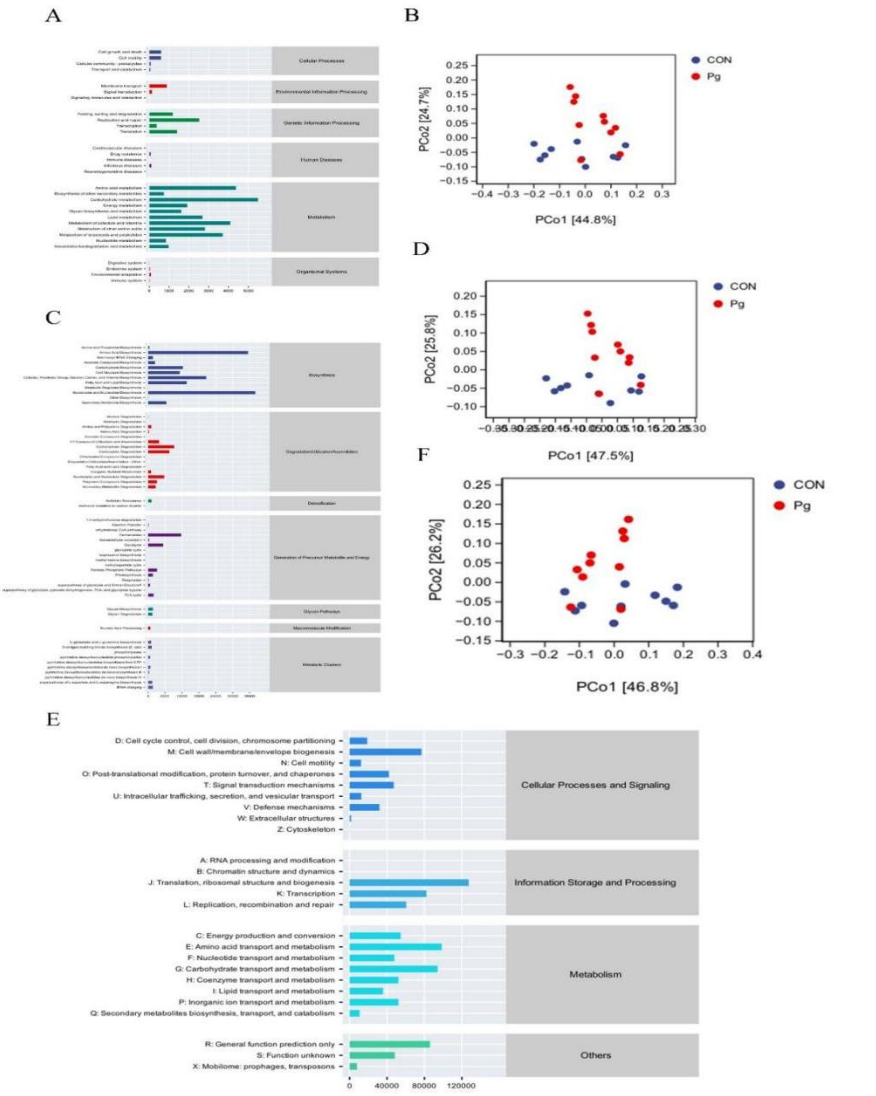

Figure S8 A: KEGG-based metabolic pathway statistics. B: KEGG functional unit PCoA analysis. C: MetaCyc-based metabolic pathway statistics. D: MetaCyc functional unit PCoA analysis. E: COG-based metabolic pathway statistics. F: COG functional unit PCoA analysis.

Detection of short-chain fatty acids

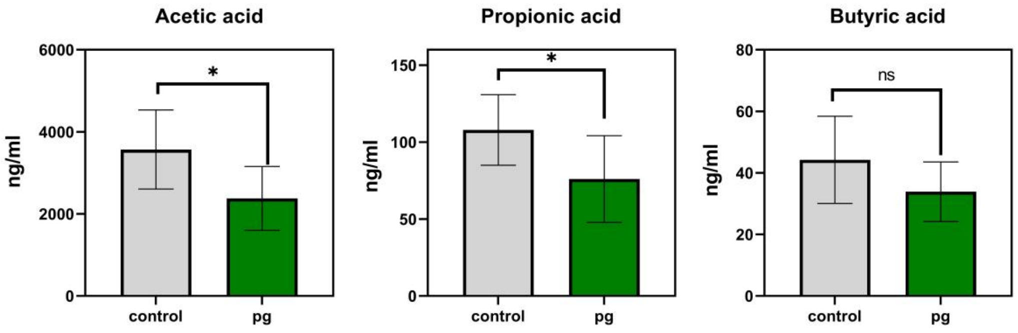

Figure S9. Different SCFA content assays

ASV distribution

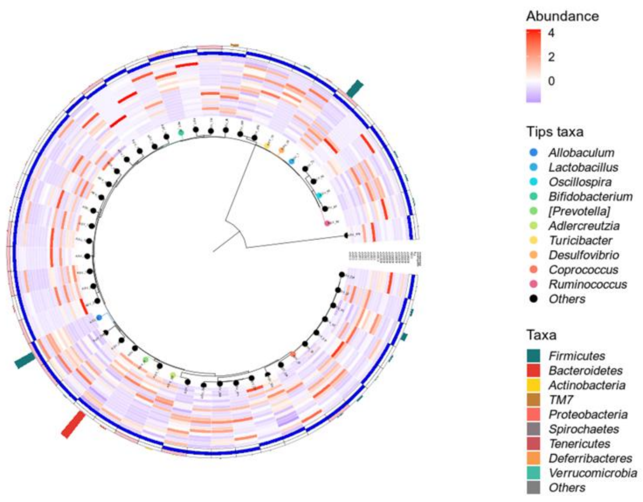

Figure S10. Phylogenetic tree plot. The main elements of the graphic include evolutionary tree diagram, heat map of abundance, differential heatmap and histogram of abundance.
